# Upregulation of interleukin-33 and thymic stromal lymphopoietin levels in the lungs of idiopathic pulmonary fibrosis

**DOI:** 10.1186/s12890-017-0380-z

**Published:** 2017-02-15

**Authors:** Jong-Uk Lee, Hun Soo Chang, Hyeon Ju Lee, Chang An Jung, Da Jeong Bae, Hyun Ji Song, Jong Sook Park, Soo-Taek Uh, Young Hoon Kim, Ki-Hyun Seo, Choon-Sik Park

**Affiliations:** 1 Department of Interdisciplinary Program in Biomedical Science Major, Soonchunhyang Graduate School, Bucheon, Korea; 2 Genome Research Center and Division of Allergy and Respiratory Medicine, Soonchunhyang University Bucheon Hospital, Bucheon, Korea; 3 Division of Respiratory and Allergy Medicine, Department of Internal Medicine, Soonchunhyang University Seoul Hospital, Seoul, Korea; 4 Division of Respiratory Medicine, Department of Internal Medicine, Soonchunhyang University Chunan Hospital, Cheonan, Korea; 5 Division of Allergy and Respiratory Medicine, Department of Internal Medicine, Soonchunhyang University Bucheon Hospital, 1174, Jung-Dong, Wonmi-Ku, Bucheon, Kyeonggi-Do 420-767 Korea

**Keywords:** IL-25, IL-33, TSLP, IPF, Innate immune response

## Abstract

**Background:**

Innate T helper type 2 (Th2) immune responses mediated by interleukin (IL)-33, thymic stromal lymphopoietin (TSLP), and IL-25 have been shown to play an important role in pulmonary fibrosis of animal models; however, their clinical implications remain poorly understood.

**Methods:**

TSLP, IL-25, and IL-33 concentrations were measured in bronchoalveolar lavage fluids obtained from normal controls (NCs; *n* = 40) and from patients with idiopathic pulmonary fibrosis (IPF; *n* = 100), non-specific interstitial pneumonia (NSIP; *n* = 22), hypersensitivity pneumonitis (HP; *n* = 20), and sarcoidosis (*n* = 19).

**Results:**

The TSLP and IL-33 levels were significantly higher in patients with IPF relative to the NCs (*p* = 0.01 and *p* = 0.0001, respectively), NSIP (*p* = 4.95E − 7 and *p* = 0.0002, respectively), HP (*p* = 0.00003 and *p* = 0.000005, respectively), and sarcoidosis groups (*p* = 0.003 and *p* = 0.0001, respectively). However, the IL-25 levels were not significantly different between NC and IPF group (*p* = 0.432). Receiver operating characteristic curves of the TSLP and IL-33 levels revealed clear differences between the IPF and NC groups (AUC = 0.655 and 0.706, respectively), as well as between the IPF and the other lung disease groups (AUC = 0.786 and 0.781, respectively). Cut-off values of 3.52 pg/μg TSLP and 3.77 pg/μg IL-33 were shown to differentiate between the IPF and NC groups with 99.2 and 94.3% accuracy. Cut-off values of 4.66 pg/μg TSLP and 2.52 pg/μg IL-33 possessed 99.4 and 93.2% accuracy for differentiating among the IPF and other interstitial lung disease groups.

**Conclusions:**

Innate immune responses may be associated with the development of IPF. Furthermore, the IL-33 and TSLP levels in BAL fluids may be useful for differentiating IPF from other chronic interstitial lung diseases.

**Electronic supplementary material:**

The online version of this article (doi:10.1186/s12890-017-0380-z) contains supplementary material, which is available to authorized users.

## Background

Idiopathic interstitial pneumonia is an umbrella term used to classify a group of lung diseases of unknown etiology characterized by the accumulation of inflammatory cells and fibrosis in the pulmonary parenchyma and interstitium. Among them, idiopathic pulmonary fibrosis (IPF) is characterized by irreversible fibrosis, resulting in gradual deterioration and poor prognosis, for which few effective therapeutics exist [1]. A variety of factors in combination with a permissive genetic background such as *MUC5B* are thought to play a role in the development of pulmonary fibrosis [2]. The initial injury process and secondary fibrosis development likely begins as the result of alveolar epithelial injury due to exposure to environmental triggers, including cigarette smoke [3], particles [4], occupational dust [5], or chemical fumes [5], radiotherapy [6], chemotherapy [6]. Endogenous triggers, include obstructive sleep apnea, chronic graft-versus-host disease and connective tissue disease followed by abnormal repair. These triggers are capable of inducing both innate and adaptive immune responses [7].

The epithelium of the respiratory tract forms a large surface area that maintains intimate contact with the environment, serving as a primary participant in innate immunity [8, 9]. IL-33, IL-25 and Thymic stromal lymphopoietin (TSLP) are the main cytokines involved in the innate immune response by the epithelium [8]. Recent studies have demonstrated a clear role for these cytokines in both the induction and amplification of Th2-mediated immunity [10–12] and lineage-negative type 2 innate lymphoid cells (ILC2s), which also produce IL-13: the dominant inducer of fibrosis in several chronic lung diseases [13].

In animal models, the lungs of bleomycin-treated mice exhibit a substantial accumulation of IL-33-positive cells [14] along with over-production of mature IL-33 by the ILC of the lungs [15]. IL-33 protein and mRNA levels are significantly higher in the BAL fluids of patients with IPF relative to healthy controls [14, 16]. In addition to IL-33, TSLP has emerged as a candidate cytokine in the pathogenesis of pulmonary fibrosis by the elevated TSLP levels seen in both systemic sclerosis [17, 18]. TSLP and its receptor are highly upregulated in IPF lung [19]. Like IL-33 and TSLP, expression of IL-25 is also increased in human IPF lung tissue and IL-25 levels were significantly increased in the BAL fluids of patients with IPF [20].

Despite the relations of the pulmonary fibrosis with IL-25, IL-33, and TSLP, the clinical implications of these cytokine remain poorly defined due to the small number of subjects (less than 15) examined in the previous studies [14, 16, 18–20]. Here, we measured the IL-25, IL-33, and TSLP levels in a relatively large cohort of patients with IPF and other interstitial lung diseases, including non-specific interstitial pneumonia (NSIP), hypersensitivity pneumonitis (HP), and sarcoidosis in order to evaluate the significance of these cytokines on clinical outcomes of IPF.

## Methods

### Study subjects

The BAL fluids were obtained from the biobank of Soonchunhyang University Hospital, Bucheon, Korea. All relevant study protocols were approved by the Ethics Committee of Soonchunhyang University Hospital (approval no. SCHBC-IRB-2015-08-25 and schbc-biobank-2015-013-01, schbc-biobank-2015-013-02). Informed written consent was obtained from each patient prior to inclusion in this study. The diagnostic criteria for IPF, NSIP, HP, and sarcoidosis were based on the international consensus statement [1, 21–23], and our previous publication [24]. All subjects were examined by physicians to obtain their medical history, followed by a chest x-ray, pulmonary function tests, high-resolution chest computed tomography (HRCT), and blood tests to exclude collagen vascular diseases. None of the patients with IPF exhibited any evidence of underlying collagen vascular diseases based on either clinical manifestations or laboratory tests. IPF was diagnosed by the presence of usual interstitial pneumonia (UIP) patterns in the pathological specimen (surgical IPF) and/or by the presence of typical clinical and HRCT features in patients not subjected to surgical lung biopsy (clinical/radiological IPF). Two pathologists examined each slide independently after being informed of the patient’s age, sex, and HCRT results.

The pathologic recognition of the NSIP pattern included two major aspects: (1) recognition of characteristic histological features; and (2) exclusion of other patterns of interstitial lung diseases (ILD) as described in the ATS/ERS 2002 classification [21], and the modified version on the histologic definition of NSIP [25]. HP was diagnosed by the presence of compatible clinical manifestations with a non-necrotizing granulomatous interstitial bronchiolocentric pneumonitis [22]. The diagnosis of sarcoidosis was made on the basis of compatible clinical pictures in combination with the histologic demonstration of non-caseating granulomas [23]. Definitive diagnosis of HP or sarcoidosis required the exclusion of other diseases capable of producing a similar histological picture, with biopsies stained using acid fast bacilli and Gömöri methenamine silver to rule out microorganisms and fungi, respectively.

All study subjects were evaluated using serial forced vital capacity (FVC) and diffusing capacity of the lungs for carbon monoxide (DLCO) measurements. The annual rate of FVC decline (dFVC%/year) was calculated as follows: (last FVC − baseline FVC)/baseline FVC/year. Healthy controls were comprised of patients’ spouses and hospital personnel with no evidence of respiratory symptoms, as determined by a screening questionnaire, a predicted FEV1 and FVC > 80%, and normal chest radiogram results.

### Bronchoalveolar lavage fluids procedure

BAL was obtained from the most significantly involved segments as determined by HRCT in the absence of immunosuppressive therapies or in the right middle lobe from NCs, as described previously [24, 26]. Total cell count was measured using a hematocytometer chamber [27]. Slides of the BAL cells prepared by cytocentrifuge were air-dried, fixed in methanol, and stained with Diff-Quik®, and a differential count of 500 cells was performed. Cell pellets were separated from the supernatants using centrifugation at 500 × *g* for 5 min, and the supernatants were stored −80 °C.

### Enzyme-linked immunosorbent assays (ELISAs) for TSLP, IL-25, and IL-33

Cytokine concentrations were measured using quantitative sandwich enzyme immunoassays for IL-25 (Bosterbio, Pleasanton, CA, USA), IL-33, and TSLP (R&D Systems, Minneapolis, MN) according to the manufacturer’s recommendations. Low detection limits were defined as 10 pg/mL IL-25, 0.52 pg/mL IL-33, and 3.46 pg/mL TSLP; values below these thresholds were recorded as 0 pg/mL. Coefficients of variance of inter- and intra-assays were <15%. Protein concentrations of BAL fluids were measured using a Micro BCA Protein Assay Kit (Pierce, Rockford, IL, USA).

### Statistical analysis

Data were analyzed using SPSS v. 20.0. The normality of the distribution was tested using the Shapiro–Wilk test. Comparisons were performed using the Kruskal–Wallis test and a post-hoc analysis; a Mann–Whitney *U* test was conducted as the nonparametric test. A receiver operating characteristic (ROC) curve, area under the curve (AUC), and a cut-off value were calculated using MedCalc [28]. Correlations between the IL-25, IL-33, and TSLP levels with the clinical outcomes were analyzed using Spearman’s correlation coefficient analysis. Values of *p* < 0.05 were considered to indicate statistical significance.

## Results

### Comparison of the clinical characteristics between the study subjects

BAL samples were obtained from subjects with IPF (*n* = 100), NSIP (*n* = 22), hypersensitivity pneumonitis (*n* = 20), and sarcoidosis (*n* = 19), along with NCs (*n* = 40) (Table 1). The IPF group consisted of 54 surgical and 46 clinical/radiological IPF patients, with no significant differences in clinical or physiological parameters between the two groups (Additional file 1: Table S1). Patients with IPF, NSIP, HP, and sarcoidosis exhibited significantly higher total cell counts, higher numbers of macrophages, neutrophils, and eosinophils in the BAL fluids, and lower FVC values relative to the controls (*p* < 0.05) (Table 1). The patients with NSIP, HP, and sarcoidosis exhibited significantly higher numbers of lymphocytes in their BAL fluids compared to those in IPF (*p* < 0.05, Table 1). Neutrophil count was significantly higher in NSIP and HP when compared with sarcoidosis (*p* < 0.05).

**Table 1:** Clinical characteristics of the study participants who underwent bronchoalveolar lavage

Items	Normal controls	IPF	NSIP	HP	Sarcoidosis
No.	40	100	22	20	19
Age (year)	55 (35–72)	63.8 (32–86)*	60.1 (39–70)	51.3 (28–70)	43.3 (28–69)
Sex (male/female)	14/26	63/37	9/13	10/10	10/9
Smoking (CS/ES/NS)	9/12/19	22/29/44	2/5/12	3/3/12	5/2/9
Follow-up duration (years)	ND	4.1 (2.1–6.3)	ND	ND	ND
FVC (% pred.)	106.1 (87.0–119)	75.0 (63.7–83.0)*	78.0 (66.0–91.8)*	64.5 (57.0–82.5)*	77.0 (65.0–86.0)*
FEV1 (% pred.)	102.1 (88.2–117)	89.0 (77.5–100.5)*	85.0 (73.8–101.3)*	74.5 (64.3–92.0)*	85.0 (64.0–101.0)*
DLCO (% pred.)	NA	64.0 (38.5–72.5)*	76.0 (59.0–92.0)*	67.0 (55.0–90.0)*	75.5 (57.8–84.5)*
dFVC (%/year)	NA	−7.0 (−16.5–0.0)	NA	NA	NA
BAL total cell count (10^4^/mL)	3.46 ± 0.82	8.83 ± 2.09*	17.64 ± 3.86*	13.03 ± 3.78*	8.45 ± 3.78*
Macrophages (10^4^/mL)	3.02 ± 0.41	7.37 ± 1.61*	11.51 ± 3.07*	8.25 ± 2.38*	6.76 ± 3.79*
Neutrophils (10^4^/mL)	0.21 ± 0.047	1.35 ± 0.40*	2.31 ± 1.01*^,^***	3.14 ± 2.36*^,^***	0.45 ± 0.16*^,^***
Eosinophils (10^4^/mL)	0.02 ± 0.006	0.48 ± 0.16*	0.45 ± 0.14*	0.41 ± 0.19*	0.11 ± 0.07*
Lymphocytes (10^4^/mL)	0.02 ± 0.006	0.17 ± 0.65*	2.68 ± 0.14*^,^**	2.20 ± 0.19*^,^**	2.17 ± 0.24*^,^**

DLCO was measured in 76 of 100 subjects with IPF, 19 of 22 subjects with NSIP, 18 of 20 subjects with HP and 17 of 19 subjects with sarcoidosis

Patient characteristics and pulmonary function test, shown as median (inter-quartile range), among the normal controls, IPF, NSIP, HP, and sarcoidosis groups were calculated with a Kruskal–Wallis analysis of variance (ANOVA) with the Mann–Whitney U as the post-hoc test. Bronchoalveolar lavage (BAL) cell numbers, shown as mean ± standard error of the mean (SEM), among the 5 groups were calculated with a one-way ANOVA and the Tukey’s honestly significant difference test as the post-hoc test

*IPF* idiopathic pulmonary fibrosis, *NSIP* non-specific interstitial fibrosis, *HP* hypersensitivity pneumonitis, *CS/ES/NS* current-smoker/ex-smoker/never-smoker, *ND* not determined, *dFVC (%)/year* annual decline rate of forced vital capacity (FVC)

Significance: compared with control: *P < 0.05, compared with IPF: **P < 0.05, compared with NSIP and HP: ***P < 0.05

### Comparison of TSLP, IL-33, and IL-25 protein levels among the groups

**Fig. 1:**
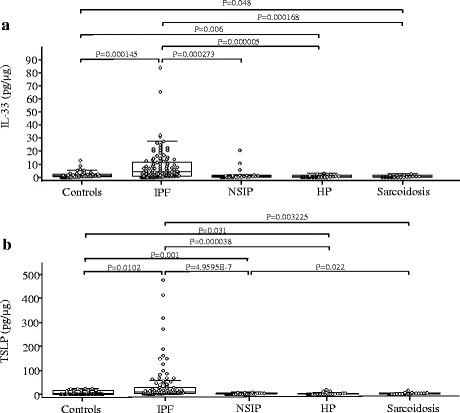
Thymic stromal lymphopoietin (TSLP) and interleukin-33 (IL-33) protein concentrations in bronchoalveolar lavage (BAL) fluid. **a** TSLP protein was detected in 23 of 40 normal controls (NCs), 100 of 100 patients with idiopathic pulmonary fibrosis (IPF), 18 of 22 patients with non-specific interstitial pneumonia (NSIP), 18 of 20 patients with hypersensitivity pneumonitis (HP), and 18 of 19 patients with sarcoidosis. The open and closed circles indicate detectable TSLP protein levels and those below the lower limit of detection (>3.46 pg/mL), respectively. **b** IL-33 protein was detected in 36 of 40 NCs, 96 of 100 patients with IPF, 20 of 22 patients with NSIP, 14 of 20 patients with HP, and 15 of 19 patients with sarcoidosis. The open and closed circles indicate detectable IL-33 protein levels and those below the lower limit of detection (>0.519 pg/mL), respectively. The data are presented as median values with the 25^th^ and 75^th^ percentiles

IL-33 was detected in 36 of 40 NCs, 96 of 100 patients with IPF, 20 of 22 patients with NSIP, 14 of 20 patients with HP, and 15 of 19 patients with sarcoidosis (Fig. 1). TSLP was detected in 23 of 40 NCs, 100 of 100 patients with IPF, 18 of 22 patients with NSIP, 18 of 20 patients with HP, and 18 of 19 patients with sarcoidosis. The median levels (interquartile ranges) of TSLP and IL-33 normalized to the total protein concentration were significantly higher in the IPF group relative to the NCs [9.27 (3.77–27.58 pg/μg) vs. 4.49 (0.31–16.04 pg/μg); *p* = 0.01, and 4.13 (0.92–12.03 pg/μg) vs. 1.15 (0.63–2.66 pg/μg); *p* = 0.0001, respectively], NSIP [1.34 (0.39–4.51 pg/μg); *p* = 4.95E − 7, and 0.82 (0.13–1.5 pg/μg); *p* = 0.0002, respectively], HP [1.42 (0.75–4.48 pg/μg); *p* = 0.00003, and 0.17 (0.31–1.73 pg/μg); *p* = 0.000005, respectively], and sarcoidosis groups [3.82 (2.0–6.53 pg/μg), *p* = 0.003, and 0.4 (0.06–1.6 pg/μg), *p* = 0.0001, respectively] (Fig. 1). The levels of IL-25 normalized to the total protein concentration were not significantly different between a subset of 24 NCs and 48 patients with IPF [8.39 (0.54–33.23) pg/μg vs. 0.0 (0.0–8.39) pg/μg, *p* = 0.432] (Additional file 2: Figure S1).

**Fig. 2:**
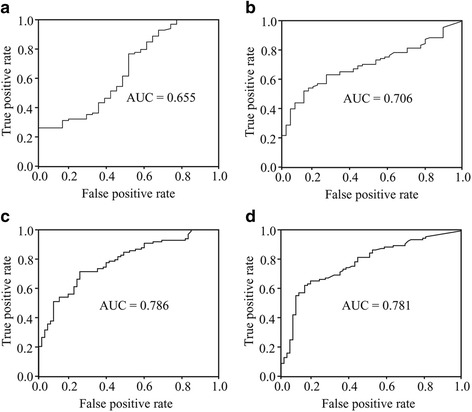
Receiver operating characteristic (ROC) curve analysis for the diagnosis of IPF. **a** The ROC curve of the TSLP protein concentration in the patients with IPF and the controls (Area under the curve [AUC] = 0.655, cut-off = 3.52 pg/μg; 99.2% accuracy, 96.7% specificity, 100% sensitivity) and **b** IL-33 protein concentration in patients with IPF and the controls (AUC = 0.706, cut-off = 3.77 pg/μg; 94.3% accuracy, 80% specificity, 100% sensitivity). **c** ROC curve of the TSLP protein concentrations among patients with IPF and those with other interstitial lung diseases (ILD) (AUC = 0.786, cut-off = 4.66 pg/μg; 99.4% accuracy, 98.4% specificity, 100% sensitivity) and **d** IL-33 protein concentration in patients with IPF and the other ILD groups (AUC = 0.781, cut-off = 2.52 pg/μg; 93.2% accuracy, 90.2% specificity, 95% sensitivity)

**Fig. 3:**
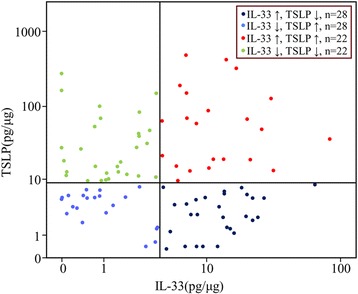
Correlation between TSLP and IL-33 levels in bronchoalveolar lavage fluid from the patients with IPF. Correlations were analyzed using Spearman’s correlation coefficient analysis. When the patients were divided into four groups using the median IL-33 (4.13 pg/μg) and TSLP (9.25 pg/μg) levels as cut-offs, 22 subjects had elevated levels of both cytokines, while 22 subjects had levels below both medians and 28 subjects had elevated levels of IL-33 only, while 28 subjects had elevated TSLP only. Differences in the clinical and physiological profiles among the four groups are presented in Table 2

The ROC curves for TSLP and IL-33 demonstrated a clear difference between the IPF and NC groups (TSLP: AUC = 0.655; IL33: AUC = 0.706) (Fig. 2a and b), as well as among the patients with IPF and those with other interstitial lung diseases (*n* = 61, TSLP: AUC = 0.786, and IL-33: AUC = 0.781) (Fig. 2c and d). Cut-off values of 3.52 pg/μg TSLP and 3.77 pg/μg IL-33 were shown to differentiate between the IPF and NC groups with 99.2 and 94.3% accuracy (96.7 and 80% specificity, respectively, with 100% sensitivity), while the cut-off values of 4.66 pg/μg TSLP and 2.52 pg/μg IL-33 possessed 99.4 and 93.2% accuracy (98.4 and 90.2% specificity and 100 and 95% sensitivity, respectively) for differentiating the IPF from the other interstitial lung disease groups. There were no evident correlations between the TSLP and IL-33 concentrations in patients with IPF (Fig. 3), nor were any correlations observed among these two cytokines and any clinical or physiological outcomes such as dFVC (%/year) or cell numbers in the BAL fluid (Additional file 1: Table S2).

When the patients were divided into 4 groups using median IL-33 (4.13 pg/μg) and TSLP (9.25 pg/μg) levels as cut-offs, 22 subjects exhibited elevated levels of both cytokines, while 22 subjects showed levels below the medians for both cytokines. A total of 28 subjects exhibited elevated levels of IL-33 only, while 28 subjects had elevated TSLP only (Fig. 3). There were no significant differences in the clinical and physiological profiles among the four groups (Table 2).

**Table 2:** Clinical characteristics of the patietns with IPF classifed according to the levels of IL-33 and TSLP

Items	IL-33↑, TSLP↓	IL-33↓, TSLP↑	IL-33↑,TSLP↑	IL-33↓, TSLP ↓
No.	28	28	22	22
Age (year)	63.5 (32–76)	67.3 (46–86)	62.5 (44–77)	62.0 (41–75)
Sex (male/female)	17/11	19/9	14 /8	13 /9
Smoking (CS/ES/NS)	7/9/12	7/8/13	2 /10 /10	10/2/7
Follow-up duration (years)	3.5 (2.28–5.53)	3.6 (2.1–5.45)	5.1 (1.6–7.2)	5.5 (2.9–7.6)
FVC (% pred.)	74.5 (61.8–84.8)	70.0 (61.5–81.8)	77.0 (65.0–86.0)	83.5 (65.5–93.0)
FEV1 (% pred.)	81.0 (75.0–100.0)	86.0 (77.0–81.8)	89.0 (79.0–102.5)	97.0 (79.5–106.3)
DLCO (% pred.)	66.0 (53.3–70.5)	46.0 (38.5–73.5)	50.0 (42.0–77.8)	68.0 (59.0–83.0)
dFVC (%)/year	−12.0 (−16.0–3.5)	−13.0 (−18.3– − 2.8)	−8.5 (−13.50– -5.75)	−10.0 (−23.5– − 0.5)
BAL total cell count (10^4^/mL)	11.27 ± 7.41	6.10 ± 1.99	5.59 ± 2.38	14.62 ± 6.07
Macrophages (10^4^/mL)	10.50 ± 6.52	5.60 ± 1.54	4.13 ± 1.25	11.19 ± 4.16
Neutrophils (10^4^/mL)	1.97 ± 1.14	1.34 ± 0.78	1.72 ± 0.92	0.47 ± 0.21
Eosinophils (10^4^/mL)	0.56 ± 0.47	0.53 ± 0.28	0.66 ± 0.42	0.12 ± 0.05
Lymphocytes (10^4^/mL)	0.43 ± 0.34	0.12 ± 0.05	0.15 ± 0.06	0.08 ± 0.28

Patient characteristics and pulmonary function test, shown as median (inter-quartile range), among the NC, IPF, NSIP, HP, and sarcoidosis groups were calculated with a Kruskal–Wallis ANOVA with the Mann–Whitney U as the post-hoc test. BAL cellular differentiation, shown as mean ± SEM, among the 4 groups was calculated with a one-way ANOVA and the Tukey’s honestly significant difference test as the post-hoc test. Significance: compared with IL-33↑:*P < 0.05

*CS/ES/NS* current-smoker/ex-smoker/never-smoker, *ND* not determined, *dFVC (%)* annual decline rate of forced vital capacity (FVC)

IL-33↑: median of IL-33 > 4.13 pg/ug, IL-33↓ ≤ 4.13 pg/ug

TSLP↑: median of TSLP > 9.25 pg/ug, TSLP↓ ≤ 9.25 pg/ug

### Comparison of TSLP, IL-33 and IL-25 protein levels among NSIP, HP, and sarcoidosis

IL-33 was slightly decreased in HP and sarcoidosis compared with NCs (0.17 (0.31–1.73 pg/μg), 0.4 (0.06–1.6 pg/μg) vs. 1.15 (0.63–2.66 pg/μg); *p* = 0.006 and *p* = 0.048, respectively). TSLP was lower in NSIP, and HP than NCs (1.34 (0.39–4.51 pg/μg), 1.42 (0.75–4.48 pg/μg) vs. 4.49 (0.31–16.04 pg/μg); *p* = 0.001, and *p* = 0.031). There was no difference of IL-33, TSLP and IL-25 levels in BAL fluids among the HP, sarcoidosis, NSIP groups (*p* > 0.05) (Fig. 1). There was no correlation of IL-33, TSLP and IL-25 levels with physiologic parameters and BAL cell counts in each group.

## Discussion

In this study, we demonstrated that IL-33 and TSLP were significantly elevated in the lungs of patients with IPF. These observations strongly support the notion of accentuated innate immune activation in the development of IPF, an effect commonly seen in animal models of acute lung injury and fibrosis. A wide variety of studies have demonstrated the importance of Th2 cells: IL-4, IL-5, and IL-13 have been causally linked to the development of fibrosis [13]. Recent studies have demonstrated a clear role of IL-33 and TSLP in both the adaptive and innate immune response. IL-13 is highly detected in the bronchoalveolar lavage (BAL) fluid of patients with IPF, and IPF fibroblasts are hyper-responsive to IL-13. Furthermore, the combined over-expression of both IL-13 and IL-13 receptor α1 directly correlates with disease severity [26]. TGF-β and IL-13 are essential for the development of pulmonary fibrosis by promoting differentiation of myofibroblast and stimulating production of extracellular matrix, such as collagens. Although IL-33, TSLP, and IL-25 might exert no direct induction of epithelial-mesenchymal transition, these cytokines are obviously involved in the process of fibrosis via regulation of TGF-β and IL-13. IL-33 polarizes M2 macrophages to produce IL-13 and TGF-β and expansion of ILC2s to producing IL-13 [15]. The interaction of these cytokines has been well documented in allergic inflammation. IL-33 stimulates the rapid expansion of type 2 innate lymphoid cells (ILC2) producing IL-13 [29], independently from the canonical CD4^+^ T helper 2 cells responses [30]. In addition to ILC2, eosinophils are additional sources of IL-13. Bone marrow-derived eosinophils secrete IL-13 in response to IL-33 stimulation in cutaneous fibrosis [31]. While IL-33 mainly affects the upstream of IL-13, TSLP acts both upstream and downstream of IL-13. TSLP induce conventional Th2 cell priming through the costimulatory action of OX40 ligand [32, 33] and an activation of ILC2 [34, 35] . In addition, TSLP is a downstream target of IL-13/Stat6 pathway [36]. Thus, the elevation of IL-33 and TSLP in IPF of our study suggest that these cytokines stimulate the upstream and downstream signals of IL-13 in concert to potentiate the process of fibrosis in the lung.

In animal models, bleomycin-mediated injury exerted a synergistic effect with IL-33 on collagen accumulation in the lung [14]. Bleomycin enhanced the production of the mature form of IL-33 in lung tissue, while deletion of the IL-33 receptor (IL-1 receptor-like 1; ST2) attenuated the bleomycin-induced lung inflammation and fibrosis [15]. Similar effects were seen following intranasal administration of a lentiviral construct expressing soluble ST2, resulting in markedly lower levels of pro-inflammatory and pro-fibrotic mediators, such as IL-4, IL-13, IL-33, and TGF-β1 along with improved survival rates in bleomycin-treated mice [37]. Although IL-33 is constitutively expressed in both epithelial and endothelial cells, IL-33 is clearly induced in the other lung tissues of patients with IPF. The infiltrating cells in and around the inflammatory lesions and the fibrotic foci, composed primarily of lymphocytes with occasional macrophages, neutrophils, and eosinophils, express IL-33 [14, 16]. In addition to the findings from animal models, elevated IL-33 levels have been observed in the BAL fluid from 10 patients with IPF compared with those from 5 healthy controls [16]. The authors also demonstrated that primary pulmonary fibroblasts derived from the lungs of the patients with IPF and systemic sclerosis, showed enhanced expression of IL-33 mRNA [16], suggesting that the fibroblasts may be an important source of IL-33.

Ever since TSLP was first implicated as a driver of Th2 responses in the airways [12], aberrant levels of TSLP have been observed in a range of airway diseases, such as asthma, COPD, and nasal polyps [38, 39]. Recently, TSLP has also emerged as an important cytokine in the pathogenesis of non-allergic diseases, including both cutaneous and lung fibrotic conditions of systemic sclerosis and IPF [17–19]. While a recent comparison of TSLP demonstrated no difference in BAL fluids between the IPF and HP groups [40], the number of subjects was too small (10 in each group) to draw any meaningful conclusions. Beyond this work, few studies have attempted to address the clinical relevance of IL-33 and TSLP expression in the context of IPF or other interstitial lung diseases. Our study clearly demonstrate that both IL-33 and TSLP levels were robustly increased in the patients with IPF compared with patients afflicted with other interstitial lung diseases, including HP, NSIP, and sarcoidosis, suggesting that the enhanced innate immune responses may be more related with the development of IPF than the other interstitial lung diseases.

Significant differences have been observed among IPF and other chronic interstitial lung diseases at both the mRNA and protein expression levels. More than 1000 genes were shown to be differentially expressed among IPF and other interstitial lung diseases, including HP and NSIP [41]; a meta-analysis of four representative datasets on IPF and sarcoidosis revealed 708 differentially expressed genes [42]. Interestingly, the expression of TSLP and IL-33 was not significantly different among the disease groups in any of these studies. The most likely explanation for the discrepancy between these mRNA expression studies is the small number of lung tissues used in the gene expression studies compared to the large number of BAL fluids in ours.

Similarly, no correlation was found between the TSLP and IL-33 concentrations in patients with IPF. This may indicate different cell sources for each of these two cytokines. IL-33 is constitutively expressed in both epithelial and endothelial cells [43], and can be expressed by several other cell types within active lesions [14, 15]. TSLP is highly expressed by lung epithelial cells [44], but is also expressed by non-epithelial cell types, including mesenchymal cells and fibrocytes. Additionally, we observed no correlations between the levels of the two cytokines and the clinical and physiological parameters, such as lung function deterioration or cell numbers in the BAL fluid. These data indicate that IL-33 and TSLP may be more likely to be related to the development of IPF rather than its severity or progression.

In the present study, IL-25 in BAL fluid was not statistically different between the patients with IPF and the normal controls. We therefore did not perform any subsequent assessments of IL-25 in other interstitial lung diseases. Pulmonary expression of IL-25 has been shown to be increased in patients with IPF, and is essential in the generation of experimental pulmonary fibrosis: IL-25 levels were shown to be higher in the BAL fluid of patients with IPF compared with a small number of controls [20]. The reason for the discrepancy between this study and ours is not clear. Prostaglandin E2 (PGE2) exhibits potent anti-fibrotic activity in the IPF lung [45]. PGE2 directly inhibits major pathobiologic functions of effector fibroblasts including chemotaxis, proliferation, collagen synthesis, and differentiation to myofibroblasts via E prostanoid receptor 2– mediated increases in intracellular cyclic AMP [46]. Derangements of PG synthesis are present in fibrotic diseases in humans and animal models of pulmonary fibrosis. Fibroblasts from IPF patients are unable to upregulate the COX-2 enzyme and are thereby deficient in PGE2 production [47]. Thus, diminished PGE2 production and/or signaling characterize lung fibrosis and are likely to be pathophysiologically significant. It is unknown whether IL-33, TSLP and IL-25 are related with PGE2 production although these cytokines stimulate mast cells to produce a large quantity of PGD2 [48]*.*

One limitation of our study was the small number of patients with other interstitial lung diseases, including those with NSIP, HP, and sarcoidosis. Additionally, long-term follow up will be necessary to fully address the clinical implications of these cytokines, particularly the survival rate of IPF. Furthermore, validation studies using other cohorts’ samples are mandatory to improve the diagnostic values of IL-33 and TSLP. Finally, the lack of transcriptome analysis in conjunction with these cytokines data limited our ability to assess some of the potential mechanisms underlying the changes in protein expression.

## Conclusions

As innate Th2-like responses have been well documented in animal models of lung fibrosis, we analyzed the clinical implications of the enhanced Th2 responses in the patients with IPF. In the present study, we demonstrated that IL-33 and TSLP, two important cytokines of the innate immune response, were elevated in the BAL fluids of the patients with IPF relative to those of the normal controls, as well as the patients with other interstitial lung diseases, including HP, NSIP, and sarcoidosis. The data presented here strongly support a role for innate immune activation in the development of IPF.

## Additional files

Additional file 1: Supplemental data. (DOC 102 kb)
Additional file 2: Figure S1. Interleukin (IL)-25 protein concentration in bronchoalveolar lavage (BAL) fluid. IL-25 protein was detected in 22 of 24 normal controls (NCs) and 44 of 48 patients with idiopathic pulmonary fibrosis (IPF). The open and closed circles indicate detectable IL-25 protein levels and those below the lower limit of detection (>10 pg/mL), respectively. The data are presented as median values with 25^th^ and 75^th^ percentiles. (TIF 2350 kb)

## Abbreviations

BAL: Bronchoalveolar lavage
HP: Hypersensitivity pneumonitis
IL-33: Interleukin-33
ILC2s: Lineage-negative type 2 innate lymphoid cells
IPF: Idiopathic pulmonary fibrosis
NSIP: Non-specific interstitial pneumonia
PGE2: Prostaglandin E2
Th1: T helper type 1
Th2: T helper type 2
TSLP: Thymic stromal lymphopoietin lineage-negative type 2 innate lymphoid cells (ILC2s)

## References

[1] American_Thoracic_Society (2000). Idiopathic pulmonary fibrosis: diagnosis and treatment. International consensus statement. American Thoracic Society (ATS), and the European Respiratory Society (ERS). Am J Respir Crit Care Med.

[2] Seibold MA, Wise AL, Speer MC, Steele MP, Brown KK, Loyd JE, Fingerlin TE, Zhang W, Gudmundsson G, Groshong SD (2011). A common MUC5B promoter polymorphism and pulmonary fibrosis. N Engl J Med.

[3] Baumgartner KB, Samet JM, Stidley CA, Colby TV, Waldron JA (1997). Cigarette smoking: a risk factor for idiopathic pulmonary fibrosis. Am J Respir Crit Care Med.

[4] Baumgartner KB, Samet JM, Coultas DB, Stidley CA, Hunt WC, Colby TV, Waldron JA (2000). Occupational and environmental risk factors for idiopathic pulmonary fibrosis: a multicenter case–control study. Collaborating Centers. Am J Epidemiol.

[5] Lee SH, Kim DS, Kim YW, Chung MP, Uh ST, Park CS, Jeong SH, Park YB, Lee HL, Song JS (2015). Association between occupational dust exposure and prognosis of idiopathic pulmonary fibrosis: a Korean national survey. Chest.

[6] Abid SH, Malhotra V, Perry MC (2001). Radiation-induced and chemotherapy-induced pulmonary injury. Curr Opin Oncol.

[7] Hoebe K, Janssen E, Beutler B (2004). The interface between innate and adaptive immunity. Nat Immunol.

[8] Parker D, Prince A (2011). Innate immunity in the respiratory epithelium. Am J Respir Cell Mol Biol.

[9] Yamamoto K, Ferrari JD, Cao Y, Ramirez MI, Jones MR, Quinton LJ, Mizgerd JP (2012). Type I alveolar epithelial cells mount innate immune responses during pneumococcal pneumonia. J Immunol.

[10] Fort MM, Cheung J, Yen D, Li J, Zurawski SM, Lo S, Menon S, Clifford T, Hunte B, Lesley R (2001). IL-25 induces IL-4, IL-5, and IL-13 and Th2-associated pathologies in vivo. Immunity.

[11] Schmitz J, Owyang A, Oldham E, Song Y, Murphy E, McClanahan TK, Zurawski G, Moshrefi M, Qin J, Li X (2005). IL-33, an interleukin-1-like cytokine that signals via the IL-1 receptor-related protein ST2 and induces T helper type 2-associated cytokines. Immunity.

[12] Zhou B, Comeau MR, De Smedt T, Liggitt HD, Dahl ME, Lewis DB, Gyarmati D, Aye T, Campbell DJ, Ziegler SF (2005). Thymic stromal lymphopoietin as a key initiator of allergic airway inflammation in mice. Nat Immunol.

[13] Wynn TA (2004). Fibrotic disease and the T(H)1/T(H)2 paradigm. Nat Rev Immunol.

[14] Luzina IG, Kopach P, Lockatell V, Kang PH, Nagarsekar A, Burke AP, Hasday JD, Todd NW, Atamas SP (2013). Interleukin-33 potentiates bleomycin-induced lung injury. Am J Respir Cell Mol Biol.

[15] Li D, Guabiraba R, Besnard AG, Komai-Koma M, Jabir MS, Zhang L, Graham GJ, Kurowska-Stolarska M, Liew FY, McSharry C (2014). IL-33 promotes ST2-dependent lung fibrosis by the induction of alternatively activated macrophages and innate lymphoid cells in mice. J Allergy Clin Immunol.

[16] Luzina IG, Pickering EM, Kopach P, Kang PH, Lockatell V, Todd NW, Papadimitriou JC, McKenzie AN, Atamas SP (2012). Full-length IL-33 promotes inflammation but not Th2 response in vivo in an ST2-independent fashion. J Immunol.

[17] Roan F, Bell BD, Stoklasek TA, Kitajima M, Han H, Ziegler SF (2012). The multiple facets of thymic stromal lymphopoietin (TSLP) during allergic inflammation and beyond. J Leukoc Biol.

[18] Usategui A, Criado G, Izquierdo E, Del Rey MJ, Carreira PE, Ortiz P, Leonard WJ, Pablos JL (2013). A profibrotic role for thymic stromal lymphopoietin in systemic sclerosis. Ann Rheum Dis.

[19] Datta A, Alexander R, Sulikowski MG, Nicholson AG, Maher TM, Scotton CJ, Chambers RC (2013). Evidence for a functional thymic stromal lymphopoietin signaling axis in fibrotic lung disease. J Immunol.

[20] Hams E, Armstrong ME, Barlow JL, Saunders SP, Schwartz C, Cooke G, Fahy RJ, Crotty TB, Hirani N, Flynn RJ (2014). IL-25 and type 2 innate lymphoid cells induce pulmonary fibrosis. Proc Natl Acad Sci U S A.

[21] American Thoracic Society/European Respiratory Society International Multidisciplinary Consensus Classification of the Idiopathic Interstitial Pneumonias (2002). This joint statement of the American Thoracic Society (ATS), and the European Respiratory Society (ERS) was adopted by the ATS board of directors, June 2001 and by the ERS Executive Committee, June 2001. Am J Respir Crit Care Med.

[22] Selman M, Pardo A, King TE (2012). Hypersensitivity pneumonitis: insights in diagnosis and pathobiology. Am J Respir Crit Care Med.

[23] Hunninghake GW, Costabel U, Ando M, Baughman R, Cordier JF, du Bois R, Eklund A, Kitaichi M, Lynch J, Rizzato G (1999). ATS/ERS/WASOG statement on sarcoidosis. American Thoracic Society/European Respiratory Society/World Association of Sarcoidosis and other Granulomatous Disorders. Sarcoidosis Vasc Diffuse Lung Dis.

[24] Park CS, Chung SW, Ki SY, Lim GI, Uh ST, Kim YH, Choi DI, Park JS, Lee DW, Kitaichi M (2000). Increased levels of interleukin-6 are associated with lymphocytosis in bronchoalveolar lavage fluids of idiopathic nonspecific interstitial pneumonia. Am J Respir Crit Care Med.

[25] Travis WD, Hunninghake G, King TE, Lynch DA, Colby TV, Galvin JR, Brown KK, Chung MP, Cordier JF, du Bois RM (2008). Idiopathic nonspecific interstitial pneumonia: report of an American Thoracic Society project. Am J Respir Crit Care Med.

[26] Park SW, Ahn MH, Jang HK, Jang AS, Kim DJ, Koh ES, Park JS, Uh ST, Kim YH, Park JS (2009). Interleukin-13 and its receptors in idiopathic interstitial pneumonia: clinical implications for lung function. J Korean Med Sci.

[27] Kim CK, Kim SW, Park CS, Kim BI, Kang H, Koh YY (2003). Bronchoalveolar lavage cytokine profiles in acute asthma and acute bronchiolitis. J Allergy Clin Immunol.

[28] Soreide K (2009). Receiver-operating characteristic curve analysis in diagnostic, prognostic and predictive biomarker research. J Clin Pathol.

[29] Barlow JL, Peel S, Fox J, Panova V, Hardman CS, Camelo A, Bucks C, Wu X, Kane CM, Neill DR (2013). IL-33 is more potent than IL-25 in provoking IL-13-producing nuocytes (type 2 innate lymphoid cells) and airway contraction. J Allergy Clin Immunol.

[30] Hung LY, Lewkowich IP, Dawson LA, Downey J, Yang Y, Smith DE, Herbert DR (2013). IL-33 drives biphasic IL-13 production for noncanonical Type 2 immunity against hookworms. Proc Natl Acad Sci U S A.

[31] Rankin AL, Mumm JB, Murphy E, Turner S, Yu N, McClanahan TK, Bourne PA, Pierce RH, Kastelein R, Pflanz S (2010). IL-33 induces IL-13-dependent cutaneous fibrosis. J Immunol.

[32] Al-Shami A, Spolski R, Kelly J, Keane-Myers A, Leonard WJ (2005). A role for TSLP in the development of inflammation in an asthma model. J Exp Med.

[33] Ito T, Wang YH, Duramad O, Hori T, Delespesse GJ, Watanabe N, Qin FX, Yao Z, Cao W, Liu YJ (2005). TSLP-activated dendritic cells induce an inflammatory T helper type 2 cell response through OX40 ligand. J Exp Med.

[34] Halim TY, Krauss RH, Sun AC, Takei F (2012). Lung natural helper cells are a critical source of Th2 cell-type cytokines in protease allergen-induced airway inflammation. Immunity.

[35] Kim BS, Siracusa MC, Saenz SA, Noti M, Monticelli LA, Sonnenberg GF, Hepworth MR, Van Voorhees AS, Comeau MR, Artis D (2013). TSLP elicits IL-33-independent innate lymphoid cell responses to promote skin inflammation. Sci Transl Med.

[36] Miyata M, Nakamura Y, Shimokawa N, Ohnuma Y, Katoh R, Matsuoka S, Okumura K, Ogawa H, Masuyama K, Nakao A (2009). Thymic stromal lymphopoietin is a critical mediator of IL-13-driven allergic inflammation. Eur J Immunol.

[37] Gao Q, Li Y, Pan X, Yuan X, Peng X, Li M (2016). Lentivirus expressing soluble ST2 alleviates bleomycin-induced pulmonary fibrosis in mice. Int Immunopharmacol.

[38] Ying S, O’Connor B, Ratoff J, Meng Q, Fang C, Cousins D, Zhang G, Gu S, Gao Z, Shamji B (2008). Expression and cellular provenance of thymic stromal lymphopoietin and chemokines in patients with severe asthma and chronic obstructive pulmonary disease. J Immunol.

[39] Kimura S, Pawankar R, Mori S, Nonaka M, Masuno S, Yagi T, Okubo K (2011). Increased expression and role of thymic stromal lymphopoietin in nasal polyposis. Allergy, Asthma Immunol. Res..

[40] Willems S, Verleden SE, Vanaudenaerde BM, Wynants M, Dooms C, Yserbyt J, Somers J, Verbeken EK, Verleden GM, Wuyts WA (2013). Multiplex protein profiling of bronchoalveolar lavage in idiopathic pulmonary fibrosis and hypersensitivity pneumonitis. Ann Thorac Med.

[41] Selman M, Pardo A, Barrera L, Estrada A, Watson SR, Wilson K, Aziz N, Kaminski N, Zlotnik A (2006). Gene expression profiles distinguish idiopathic pulmonary fibrosis from hypersensitivity pneumonitis. Am J Respir Crit Care Med.

[42] Leng D, Huan C, Xie T, Liang J, Wang J, Dai H, Wang C, Jiang D (2013). Meta-analysis of genetic programs between idiopathic pulmonary fibrosis and sarcoidosis. PLoS One.

[43] Moussion C, Ortega N, Girard JP (2008). The IL-1-like cytokine IL-33 is constitutively expressed in the nucleus of endothelial cells and epithelial cells in vivo: a novel ‘alarmin’?. PLoS One.

[44] Soumelis V, Reche PA, Kanzler H, Yuan W, Edward G, Homey B, Gilliet M, Ho S, Antonenko S, Lauerma A (2002). Human epithelial cells trigger dendritic cell mediated allergic inflammation by producing TSLP. Nat Immunol.

[45] Park GY, Christman JW (2006). Involvement of cyclooxygenase-2 and prostaglandins in the molecular pathogenesis of inflammatory lung diseases. Am J Physiol Lung Cell Mol Physiol.

[46] Huang S, Wettlaufer SH, Hogaboam C, Aronoff DM, Peters-Golden M (2007). Prostaglandin E(2) inhibits collagen expression and proliferation in patient-derived normal lung fibroblasts via E prostanoid 2 receptor and cAMP signaling. Am J Physiol Lung Cell Mol Physiol.

[47] Wilborn J, Crofford LJ, Burdick MD, Kunkel SL, Strieter RM, Peters-Golden M (1995). Cultured lung fibroblasts isolated from patients with idiopathic pulmonary fibrosis have a diminished capacity to synthesize prostaglandin E2 and to express cyclooxygenase-2. J Clin Invest.

[48] Buchheit KM, Cahill KN, Katz HR, Murphy KC, Feng C, Lee-Sarwar K, Lai J, Bhattacharyya N, Israel E, Boyce JA (2016). Thymic stromal lymphopoietin controls prostaglandin D2 generation in patients with aspirin-exacerbated respiratory disease. J Allergy Clin Immunol.

