# Supplementary material for: Upregulation of interleukin-33 and thymic stromal lymphopoietin levels in the lungs of idiopathic pulmonary fibrosis
Source: BMC Pulm Med. 2017 Feb 15;17:39. doi: 10.1186/s12890-017-0380-z (PMC5312598; doi:10.1186/s12890-017-0380-z)
Supplement: Additional file 1: — Supplemental data. (DOC 102 kb) [file 12890_2017_380_MOESM1_ESM.doc]

**Additional file 1**

Table S1. Clinical characteristics of the patietns with surgical and those with clinical IPF

| Items | Clinical/Radiological IPF | Surgical |
| --- | --- | --- |
| No. | 46 | 54 |
| Age (year) | 61.0 (41–76) | 66.0 (32–86) |
| Sex (male/female) | 25/21 | 38/16 |
| Smoking (CS/ES/NS) | 6/20/23 | 16/9/21 |
| Survival/Death | 7/3 | 19/7 |
| Follow-up duration (years) | 4.2 (1.9–8.0) | 3.8 (2.1–5.6) |
| FVC (% pred.) | 75.5 (60.3–85.5) | 73.0 (65.0–84.8) |
| FEV1 (% pred.) | 85.5 (75.0–99.0) | 90.0 (79.0–104.0) |
| DLCO (% pred.) | 65.0 (43.3–73.0) | 65.0 (40.8–73.0) |
| dFVC (%)/year | −12.0 (−19.0– −2.0) | −4.0 (−15.0–0.5) |
| BAL total cell count (10^4^/mL) | 10.47 ± 3.58 | 7.12 ± 2.14 |
| Macrophages (10^4^/mL) | 9.02 ± 2.81 | 5.67 ± 1.47 |
| Neutrophils (10^4^/mL) | 1.43 ± 0.62 | 1.26 ± 0.51 |
| Eosinophils (10^4^/mL) | 0.36 ± 0.17 | 0.59 ± 0.28 |
| Lymphocytes (10^4^/mL) | 0.09 ± 0.03 | 0.24 ± 0.12 |
| IL-33(pg/µg) | 4.98(1.03-13.93) | 3.92(0.92-8.62) |
| TSLP(pg/µg) | 7.46(3.66-19.02) | 10.79(3.94-24.85) |

Patient characteristics and pulmonary function test, shown as median (inter-quartile range), among the controls, idiopathic pulmonary fibrosis (IPF), non-specific interstitial pneumonia (NSIP), hypersensitivity pneumonitis (HP), and sarcoidosis groups were calculated with a Mann–Whitney U test. Bronchoalveolar lavage (BAL) cellular differentiation, shown as mean ± standard error of the mean (SEM), among the groups were calculated with a *t*-test. CS/ES/NS: current-smoker/ex-smoker/never-smoker, dFVC(%)/year: annual decline rate of forced vital capacity (FVC).

Table S2. Correlation of Thymic stromal lymphopoietin (TSLP) and interleukin (IL)-33 levels with cell profiles from the BAL fluid and the lung function profiles in IPF.

| Correlations | N | TSLP | |  | IL-33 | |
| --- | --- | --- | --- | --- | --- | --- |
|  |  | Correlation coefficient | P-value |  | Correlation coefficient | P-value |
| Macrophage (count) | 84 | −0.048 | 0.636 |  | −0.080 | 0.433 |
| Lymphocyte (count) | 84 | 0.015 | 0.888 |  | −0.123 | 0.242 |
| Neutrophil (count) | 84 | 0.078 | 0.509 |  | 0.118 | 0.318 |
| Eosinophil (count) | 84 | 0.096 | 0.394 |  | 0.184 | 0.099 |
| FVC% | 98 | −0.072 | 0.515 |  | 0.015 | 0.889 |
| FEV1% | 93 | −0.059 | 0.595 |  | 0.018 | 0.871 |
| dFVC%/year | 74 | −0.040 | 0.718 |  | 0.120 | 0.276 |
| DLCO% | 76 | −0.131 | 0.236 |  | 0.034 | 0.756 |

dFVC%/year: Annual rate of FVC decline was calculated as follows: (last FVC − baseline FVC)/baseline FVC/year. Correlations between the IL-25, IL-33, and TSLP levels with the clinical outcomes were analyzed using Spearman’s correlation coefficient analysis. Values of p < 0.05 were considered to indicate statistical significance.

Table S3. Clinical characteristics and cytokine levels of the patients with fibrosing and those with cellular NSIP

| Items | NSIP | | p-value |
| --- | --- | --- | --- |
|  | Fibrosing | Cellular |  |
| No. | 19 | 3 |  |
| FVC (% pred.) | 68(65-74.5) | 73(57-81.5) | 0.723 |
| FEV1 (% pred.) | 76(66.5-84.5) | 85(61-97) | 0.842 |
| DLCO (% pred.) | 68.5(64.25-72.75) | 62(49-75) | 0.657 |
| BAL total cell count (10^4^/mL) | 15.02±19.03 | 18.9±3.75 | 0.667 |
| Macrophages (10^4^/mL) | 10.84±0.89 | 12.8±1.66 | 0.665 |
| Neutrophils (10^4^/mL) | 2.23±1.11 | 2.64±1.38 | 0.05 |
| Eosinophils (10^4^/mL) | 0.39±0.3 | 0.51±0.22 | 0.312 |
| Lymphocytes (10^4^/mL) | 2.53±0.55 | 2.75±0.23 | 0.924 |
| IL-33(pg/µg) | 0.71(0.17-1.42) | 1.09(0.58-3.45) | 0.666 |
| TSLP(pg/µg) | 1.79(0.27-4.51) | 1.32(1.05-1.33) | 0.737 |

Differences in patient characteristics [shown as medians (interquartile ranges)] and between the subgroups of NSIP were evaluated using the Mann–Whitney U-test. Differences in patient characteristics [shown as means ± standard error of the mean (SEM)] among the subgroups were evaluated by t-test.

Table S4. Clinical characteristics and cytokine levels of the patients with subacute and those with chronic HP

| Items | HP | | p-value |
| --- | --- | --- | --- |
|  | Subacute | Chronic |  |
| No. | 17 | 3 |  |
| FVC (% pred.) | 63(58.5-73) | 71(56-81) | 0.895 |
| FEV1 (% pred.) | 75(68-76.5) | 79(65-93) | 0.467 |
| DLCO (% pred.) | 60(54.5-75) | 67(54.5-74) | 0.948 |
| BAL total cell count (10^4^/mL) | 12.66±15.67 | 16.39±3.8 | 0.921 |
| Macrophages (10^4^/mL) | 7.67±3.69 | 10.5±1.85 | 0.322 |
| Neutrophils (10^4^/mL) | 3.16±2.72 | 3.11±1.47 | 0.189 |
| Eosinophils (10^4^/mL) | 0.17±0.59 | 0.54±0.18 | 0.334 |
| Lymphocytes (10^4^/mL) | 2.25±0.36 | 2.48±0.28 | 0.130 |
| IL-33(pg/µg) | 0.26(0.00-0.92) | 2.32(1.16-2.73) | 0.334 |
| TSLP(pg/µg) | 1.51(0.82-4.66) | 0.75(0.49-2.35) | 0.368 |

Differences in patient characteristics [shown as medians (interquartile ranges)] and between the subgroups of HP were evaluated using the Mann–Whitney U-test. Differences in patient characteristics [shown as means ± standard error of the mean (SEM)] among the subgroups were evaluated by t-test.

Table S5. Clinical characteristics and cytokine levels of the patients with sarcoidosis according to the stages

| Items | Sarcoidosis | | | p-value |
| --- | --- | --- | --- | --- |
|  | 1 | 2 | 3 |  |
| No. | 7 | 9 | 3 |  |
| FVC (% pred.) | 91(79.5-95.5) | 79(75.5-85) | 81(69.5-90) | 0.501 |
| FEV1 (% pred.) | 100(89.5-109) | 82(76.25-92) | 96(78-101.5) | 0.301 |
| DLCO (% pred.) | 86.5(79-86.125) | 89.5(83-98.5) | 87(69.5-88.5) | 0.309 |
| BAL total cell count (10^4^/mL) | 9.03±6.19 | 9.88±7.9 | 4.14±3.93 | 0.507 |
| Macrophages (10^4^/mL) | 6.82±2.29 | 6.95±2.4 | 6.51±1.16 | 0.931 |
| Neutrophils (10^4^/mL) | 0.45±0.87 | 0.65±0.78 | 0.3±0.77 | 0.615 |
| Eosinophils (10^4^/mL) | 0.08±0.04 | 0.65±0.54 | 0.9±0.84 | 0.650 |
| Lymphocytes (10^4^/mL) | 2.67±0.36 | 2.48±0.7 | 1.95±0.82 | 0.302 |
| IL-33(pg/µg) | 0.23(0.03-0.53) | 1.60(0.43-1.86) | 0.06(0.03-0.37) | 0.172 |
| TSLP(pg/µg) | 3.81(2.01-4.93) | 4.20(2.00-6.53) | 2.87(2.78-19.58) | 0.889 |

Differences in patient characteristics [shown as medians (interquartile ranges)] and between the subgroups of sarcoidosis were evaluated using the Mann–Whitney U-test. Differences in patient characteristics [shown as means ± standard error of the mean (SEM)] among the subgroups were evaluated by t-test.
