# Supplementary figures and images for: Upregulation of interleukin-33 and thymic stromal lymphopoietin levels in the lungs of idiopathic pulmonary fibrosis
Source: BMC Pulm Med. 2017 Feb 15;17:39. doi: 10.1186/s12890-017-0380-z (PMC5312598; doi:10.1186/s12890-017-0380-z)

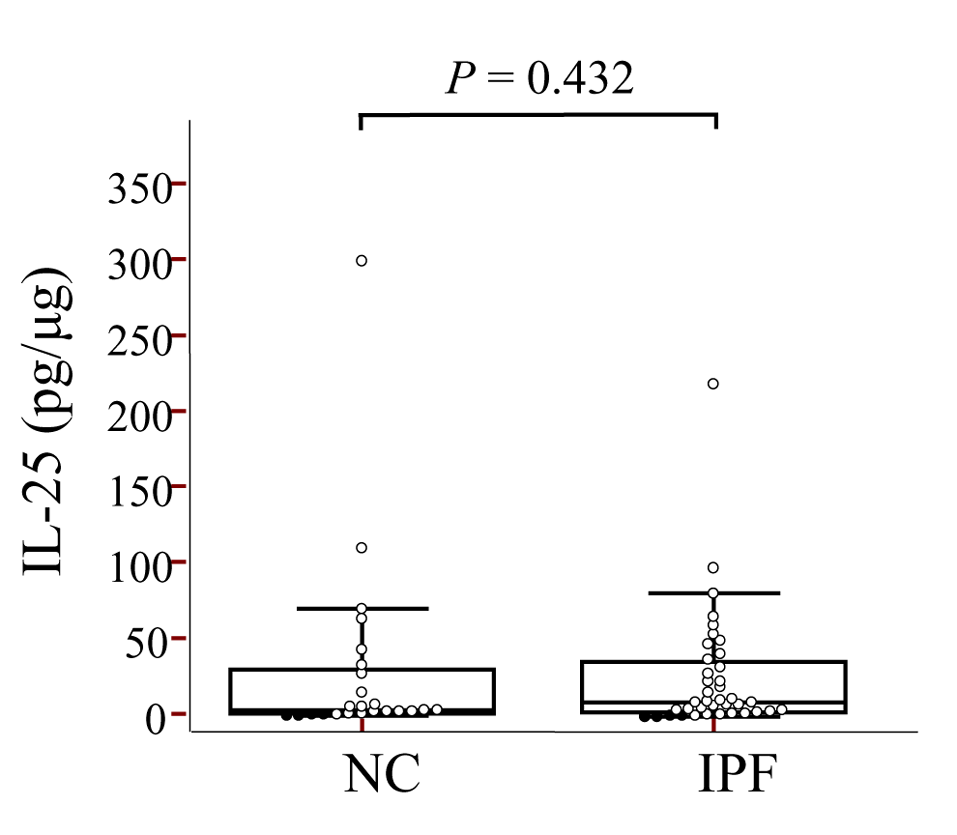

Supplement: Additional file 2: Figure S1. — Interleukin (IL)-25 protein concentration in bronchoalveolar lavage (BAL) fluid. IL-25 protein was detected in 22 of 24 normal controls (NCs) and 44 of 48 patients with idiopathic pulmonary fibrosis (IPF). The open and closed circles indicate detectable IL-25 protein levels and those below the lower limit of detection (>10 pg/mL), respectively. The data are presented as median values with 25th and 75th percentiles. (TIF 2350 kb) [file 12890_2017_380_MOESM2_ESM.tif]
